# A173 PATTERNS OF FISTULA DISEASE ACTIVITY IN PATIENTS WITH PERIANAL CROHN’S DISEASE

**DOI:** 10.1093/jcag/gwae059.173

**Published:** 2025-02-10

**Authors:** N Huebener, A Sweet, J McCurdy

**Affiliations:** Faculty of Medicine, University of Ottawa Faculty of Medicine, Ottawa, ON, Canada; Faculty of Medicine, University of Ottawa Faculty of Medicine, Ottawa, ON, Canada; Faculty of Medicine, University of Ottawa Faculty of Medicine, Ottawa, ON, Canada

## Abstract

**Background:**

The disease course of perianal fistulizing Crohn’s disease (PFCD) is highly variable, from transient symptoms to chronic, progressive disease activity. The patterns of PFCD activity over time and their impact on long-term fistula outcomes is poorly understood.

**Aims:**

To determine the patterns of PFCD activity within the first year of fistula diagnosis and their impact long-term outcomes.

**Methods:**

We performed a retrospective, observational study between 2005-2023 at a single Canadian academic institution. We included adults >17 years with PFCD and a minimum of 2 clinical touchpoints within the first year after fistula diagnosis. PFCD was categorized into one of four patterns of disease activity: transient, minimally active, relapsing-remitting, and chronic persistent based on clinical symptoms within the first year after fistula diagnosis (Figure 1). Our primary outcome was major adverse fistula outcomes (MAFO), a composite of local surgery, hospitalization, and fecal diversion for PFCD. Secondary outcomes included individual components of MAFO and fistula remission at last follow-up. Time to event analyses were estimated by Kaplan Meir methods stratified by disease activity categories.

**Results:**

101 patients met our study criteria. Mean age at PFCD diagnosis was 35 (SD=13) years, 46 (46%) patients were female, and 76 (78%) had complex fistula anatomy radiologically. A total of 44 (44%) patients underwent local surgical intervention at the time of diagnosis and 92 (91%) patients were treated with an advanced medical therapy. Patients were classified as having transient (n=35, 34%), minimally active (n=17, 17%), relapsing remitting (n=13, 13%) and chronic persistent (n=36, 36%) disease. After a mean follow-up of 114 months (SD=61.4), 65 (64%) of patients developed MAFO. At last follow-up 62 (61%) of patients were in remission. Table 1 shows long-term fistula outcomes stratified by each of the four disease activity categories. The proportion of patients with MAFO over time was lowest in the transient disease category. Fistula remission at last follow-up was highest in the transient disease category and lowest in the chronic persistent disease category.

**Conclusions:**

Our study shows that PFCD can be categorized into 4 distinct disease activity categories within the first year of diagnosis and that these patterns may predict long-term fistula outcomes.

Table 1: PFCD outcomes according to clinical disease activity pattern

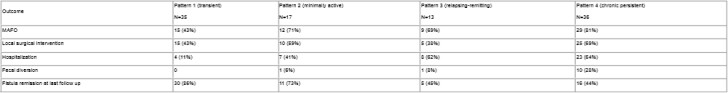

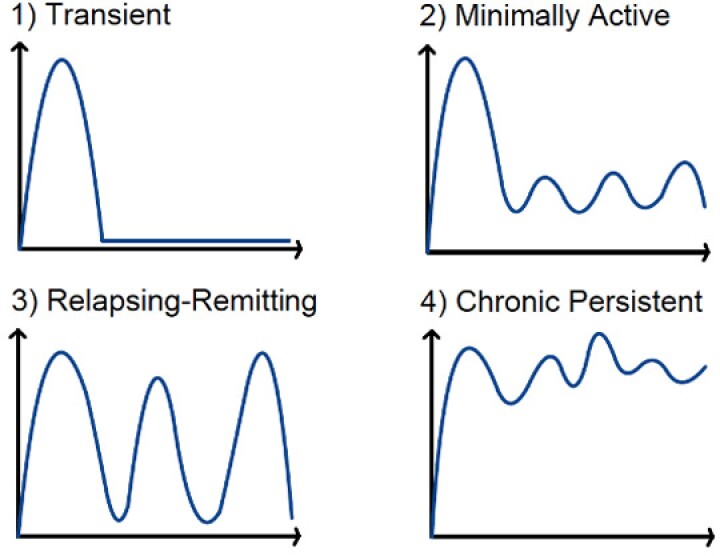

Figure 1: Patterns of PFCD Activity

**Funding Agencies:**

None

